# Concordance Between Biochemical and Molecular Diagnosis Obtained by WES in Mexican Patients with Inborn Errors of Intermediary Metabolism: Utility for Therapeutic Management

**DOI:** 10.3390/ijms252111722

**Published:** 2024-10-31

**Authors:** Marcela Vela-Amieva, Miguel Angel Alcántara-Ortigoza, Ariadna González-del Angel, Liliana Fernández-Hernández, Miriam Erandi Reyna-Fabián, Bernardette Estandía-Ortega, Sara Guillén-López, Lizbeth López-Mejía, Leticia Belmont-Martínez, Rosa Itzel Carrillo-Nieto, Isabel Ibarra-González, Seung-Woo Ryu, Hane Lee, Cynthia Fernández-Lainez

**Affiliations:** 1 Laboratorio de Errores Innatos del Metabolismo y Tamiz, Instituto Nacional de Pediatría, Secretaría de Salud, Mexico City C.P. 04530, Mexico; 2 Laboratorio de Biología Molecular, Instituto Nacional de Pediatría, Secretaría de Salud, Mexico City C.P. 04530, Mexico; 3 Unidad de Genética de la Nutrición, Instituto de Investigaciones Biomédicas, UNAM, Mexico City C.P. 04530, Mexico; 4 3billion, Inc., Seoul 03161, Republic of Korea

**Keywords:** whole-exome sequencing, diagnostic odyssey, inborn errors of metabolism, genomic medicine, personalized medicine, precision medicine, rare diseases

## Abstract

Biochemical phenotyping has been the milestone for diagnosing and managing patients affected by inborn errors of intermediary metabolism (IEiM); however, identifying the genotype responsible for these monogenic disorders greatly contributes to achieving these goals. Herein, whole-exome sequencing (WES) was used to determine the genotypes of 95 unrelated Mexican pediatric patients suspected of having IEiM. They were classified into those bearing specific biochemical abnormalities (Group 1), and those presenting unspecific biochemical profiles (Group 2). The overall concordance between the initial biochemical diagnosis and final genotypic diagnoses was 72.6% (*N* = 69/95 patients), with the highest concordance achieved in Group 1 (91.3%, *N* = 63/69), whereas the concordance was limited in Group 2 (23.07%). This finding suggests that previous biochemical phenotyping correlated with the high WES diagnostic success. Concordance was high for urea cycle disorders (94.1%) and organic acid disorders (77.4%). The identified mutational spectrum comprised 83 IEiM-relevant variants (pathogenic, likely pathogenic, and variants of uncertain significance or VUS), including three novel ones, distributed among 29 different genes responsible for amino acid, organic acid, urea cycle, carbohydrate, and lipid disorders. Inconclusive WES results (7.3%, *N* = 7/95) relied on monoallelic pathogenic genotypes or those involving two VUS for autosomal-recessive IEiMs. A second monogenic disease was observed in 10.5% (*N* = 10/95) of the patients. According to the WES results, modifications in treatment had to be made in 33.6% (*N* = 32/95) of patients, mainly attributed to the presence of a second monogenic disease, or to an actionable trait. This study includes the largest cohort of Mexican patients to date with biochemically suspected IEiM who were genetically diagnosed through WES, underscoring its importance in medical management.

## 1. Introduction

High-performance genetic testing involving gene panels, whole-exome sequencing (WES), or whole-genome sequencing (WGS) has emerged as a pivotal tool in Mendelian disease diagnostics [1]. Their expanding utility is notably evident in the precise diagnosis of critically ill children admitted to intensive care units [2,3] and has significantly reduced the diagnostic odyssey associated with rare diseases [4,5]; thus, these tests are expected to be routinely incorporated into pediatric medical care [6]. Variable diagnostic yields for suspected inborn errors of metabolism (IEM) and neurogenetic disorders using next-generation sequencing (NGS) technologies range from 16% to 68% [6,7], revealing wide differences among diverse populations and the employed clinical approaches [8]. Moreover, these diagnostic strategies are related to high rates of medical treatment redirection. For example, Wu et al. recommended specific medications or modifications for the clinical management of 45.5% and 81%, respectively, of studied patients after they reached a molecular diagnosis [9]. Other authors have shown that at least one medical management change related to the application of rapid WES was implemented in 52% of critically ill children [10]. In particular, genetic testing is essential in the diagnostic approach of rare diseases, such as IEM, which are monogenic disorders that involve abnormalities in enzymes, transport proteins, or chaperones [11], as well as to delineate or expand the genotypic and phenotypic spectrum underlying these diseases, and even to redirect medical, nutritional, surgical, or palliative management [12,13]. Genetic testing is also considered essential for prescribing some genotype-dependent IEM treatments (e.g., tyrosinemia type I, phenylketonuria, cystic fibrosis, and tetrahydrobiopterin defects) [14,15]. Additionally, WES/WGS can identify the carrier status or the co-occurrence of other monogenic traits [16,17].

Inborn errors of intermediary metabolism (IEiM) are a subgroup of IEM that comprises defects disrupting the metabolic pathways of proteins, carbohydrates, or lipids, leading to the accumulation of toxic substances or deficiency of essential compounds [11]. Before the wide availability of NGS, the cornerstone of the diagnosis of IEiM patients relied on biochemical measurements of their characteristic metabolites in blood and/or urine, which allowed us to establish a diagnosis, i.e., the elevated blood concentration of branched-chain amino acids along with alloisoleucine, and abnormal excretion of urinary alfa-ketoacids are indicative of maple syrup urine disease (MSUD) [18]; however, not all IEiMs can be unequivocally diagnosed by biochemical profiles [19,20,21]. Thus, the advent of NGS has contributed to establishing a definitive diagnosis, especially in patients whose biochemical profile is unspecific, i.e., high blood concentrations of hydroxy-isovalerylcarnitine (C5OH) in patients with seizures, hyperlactatemia, hypoglycemia with a normal acylcarnitine profile, or such cases whose clinical picture is highly suggestive of IEiM but whose biochemical profile is negative [22]. 

Unfortunately, in low- or middle-income countries, the possibility of performing genetic testing is not available for all patients; for example, we previously reported that only 33.4% of Mexican patients with an IEiM who were admitted to our tertiary referral hospital had access to diagnostic genetic testing, leading to limited data on the genotypic spectrum underlying these rare diseases in our population [23]. Thus, strategies must be designed and implemented in these countries to diminish the diagnostic gap with other high-income countries [24]. Furthermore, to the best of our knowledge, there are still no reports regarding the usefulness of WES analysis in the diagnostic approach of IEiM among Latin American patients. 

Herein, we present the results of WES in 95 patients either with biochemically confirmed IEiM or with the clinical and biochemical suspicion of having an uncharacterized IEiM to determine the following: (a) the concordance between the initial biochemical diagnosis and the responsible genotype identified by WES, (b) the genotypic spectrum underlying the IEiM in the studied Mexican patients, (c) the proportion of patients affected by a second monogenic disease (co-occurrence) due to expected, incidental, or secondary findings, and (d) the modifications in medical or nutritional management after WES results in selected cases.

## 2. Results

### 2.1. Study Population and WES Diagnostic Yield

**Figure 1:**
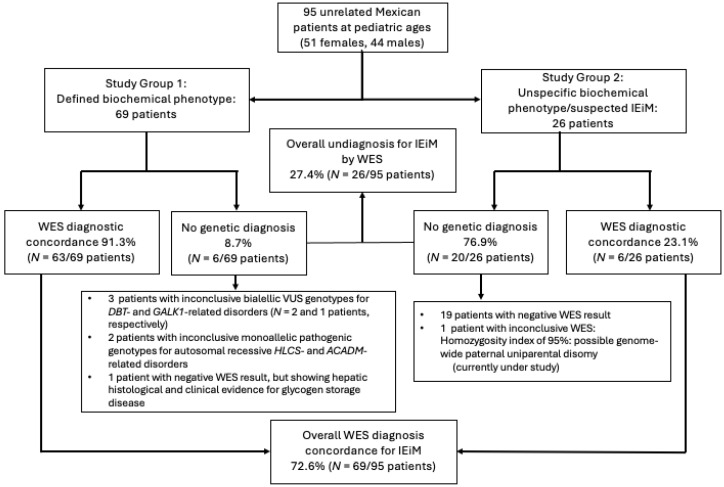
Overall, WES diagnosis concordance of the entire study population and by study groups. Note that maximum concordance was reached for those patients bearing a well-defined biochemical phenotype (Group 1) for a specific IEiM (91.3%). Instead, only in 23.1% of patients bearing an unspecific biochemical alteration (Group 2) suggesting an underlying IEiM did WES achieve a diagnostic genotype of a specific IEiM. Abbreviations: IEiM, inborn errors of intermediary metabolism; WES, whole-exome sequencing; VUS, variant of uncertain significance; DBT, branched-chain acyl transferase E2 component; GALK1, galactokinase-1 deficiency; HLCS, holocarboxylase synthetase; ACADM, acyl-CoA dehydrogenase medium-chain.

Study Group 1 included 69 patients (38 females, and 31 males, mean age ± SD was 10.9 ± 7.4 years) with a well-defined biochemical phenotype indicative of a specific IEiM (Figure 1). Moreover, study Group 2 comprised 26 patients (13 females, 13 males, mean age ± SD was 9.5 ± 6.8 years) with nonspecific alterations suggestive of an IEiM. The overall percentage and number of patients, as well as those classified according to the categorization of IEiM type, are shown in Figure 1. Consanguinity was found in 14.73% (*N* = 14/95) of the families, and endogamy was documented in 23.15% (*N* = 22/95) of them. 

**Figure 2:**
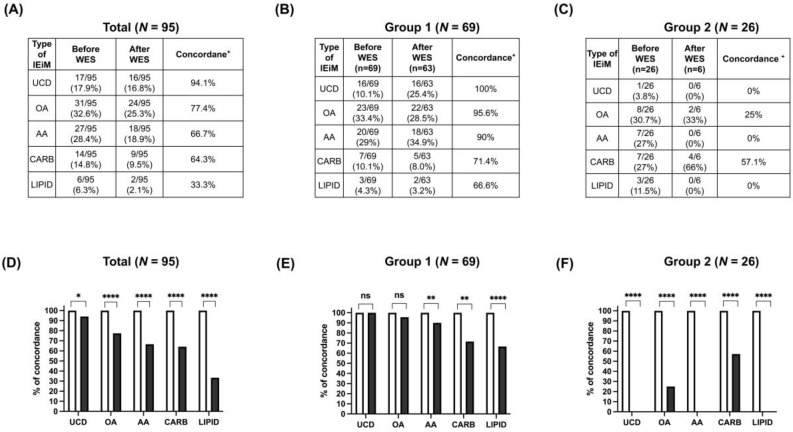
Concordance between the initial biochemical diagnosis and WES results performed in 95 patients by type of disorder: (**A**) total, (**B**) Group 1, and (**C**) Group 2. Biochemically confirmed or suspected IEiM categories in the studied population by type of IEiM and by group before (light bars) and after WES (dark bars) in the overall study population (**D**), Group 1 (**E**), and Group 2 (**F**). Concordance**^+^** between the initial and final diagnoses is the degree to which the initial biochemical diagnosis matches the final molecular diagnosis. Abbreviations: UCD, urea cycle disorders; OA, organic acid disorders; AA, amino acid disorders; CARB, carbohydrate disorders; LIPID, lipid defects. A *p*-value < 0.05 was considered to be statistically significant (* *p* < 0.05, ** *p* < 0.01, and **** *p* < 0.0001, ns: not statistically significant).

The overall concordance between the initial biochemical diagnosis and WES results was 72.6% (*N* = 69/95), with a proportion of 91.3% (*N* = 63/69) in Group 1, and 23.1% (*N* = 6/26) in Group 2 (Figure 1). The concordance by study group and by type of disorder are shown in Figure 2A–F. Notably, urea cycle disorders had the highest overall concordance between initial and final diagnosis (94.1%), followed by organic acid (77.4%), amino acid (66.6%), carbohydrate (64.2%), and lipid disorders (33.4%; Figure 2A). In Group 1, patients with urea cycle disorders presented a WES diagnostic yield of 100%. Conversely, the highest WES diagnostic yield in Group 2 was observed in patients with carbohydrate disorders, representing 66%. Overall, the comparisons between the initial biochemical and final WES diagnoses were statistically different (Figure 2D). This was different when comparing between types of disorder in Group 1 since statistical differences were only observed in amino acids, organic acids, carbohydrates, and lipid disorders (Figure 2E). In Group 2, the comparison between types of disorder was statistically different for all of them (Figure 2F).

### 2.2. The Genotypic Spectrum of IEiM-Positive Cases

In this study, we identified 83 IEiM-relevant variants distributed among 29 different genes, with pathogenic variants being the most commonly found (Table 1). 

All of them were already submitted by us to the Leiden Open Variation Database (LOVD) v.3.0 Build 30 (https://www.lovd.nl/, accessed on 21 June 2024). Three novel variants were found: NM_000159.4:c.1173_1174insT, or p.(Asn392Ter), in *GCDH*, NM_001370658.1:c.1352G>C, or p.(Cys451Ser), in *BTD*, and NM_005271.5:c.1466C>G, or p.(Pro489Arg), in *GLUD1*. The detailed information, classification by pathogenicity, and allele frequency are shown in Supplementary Table S1. Missense variants were the most common among organic acid (*N* = 18/29; 62%), urea cycle (*N* = 10/17; 58.8%), carbohydrate (*N* = 10/14; 71.4%), and lipid disorders (*N* = 2/4; 50%), whereas indel variants were the most common in amino acid disorders (7/19; 36.8%). Homozygosis was found in 32 of the 64 different identified genotypes (50%; Table 2). 

**Figure 3:**
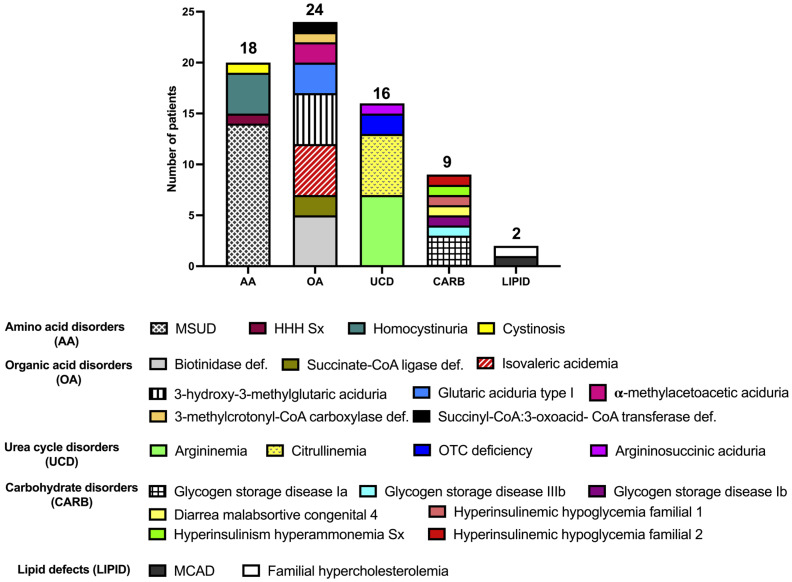
WES-positive IEiM landscape found in the studied Mexican patients. Abbreviations:CoA: coenzyme A; Def: deficiency; HHH Sx: hyperornithinemia-hyperammonemia-homocitrullinuria syndrome; MCAD: medium-chain acyl-CoA dehydrogenase deficiency, MSUD: maple syrup urine disease; OTC: ornithine transcarbamylase.

The highest number of diagnostic genotypes was documented in carbohydrate (9 genotypes in 9 patients, 100%) and lipid disorders (2 genotypes in 2 patients, 100%), followed by organic acid (23 genotypes in 24 patients, 96%), urea cycle (14 genotypes in 16 patients, 87.5%), and amino acid (15 genotypes in 18 patients, 83%) disorders. The WES-positive IEiM landscape reported in the studied population is shown in Figure 3. 

### 2.3. Unsolved Cases

This category included negative (*N* = 20/95) and inconclusive (*N* = 6/95) patients, representing 27.4% (*N* = 26/95) of the included patients, most of whom (*N* = 20/26) belonged to Group 2 (Figure 1). The primary biochemical biomarkers related to these unsolved cases are shown in Table 3, which highlights that in Group 1, branched-chain amino acids and acylcarnitines were the most common ones (*N* = 2/6 each, 33.4%). In contrast, in Group 2, acylcarnitines, especially long-chain ones (C16, C18, and C18:1), were the initial biomarkers in 35% of them (*N* = 7/20 patients). 

### 2.4. Patients with Co-Occurrence of Other Monogenic Diseases

A second monogenic disease was identified in 10.5% (*N* = 10/95) of our studied population (Table 4). The expected findings were identified in 4/10 patients, as their previous biochemical profile or clinical phenotype strongly suggested the presence of a second monogenic trait. These four patients presented postaxial polydactyly (3bINP-021), craniosynostosis (3bINP-054), cystinosis with unexplainable and progressive renal failure despite adequate cysteamine treatment (3bINP-082), and G6PD deficiency (3bINP-085), which were confirmed when pathogenic variants were identified in the *GLI3*, *FGFR2*, *COL4A5*, and *G6PD* genes, respectively (Table 4). 

Three patients revealed incidental findings in the *ABCA4* (3bINP-069), *PIKFYVE* (3bINP-109), and *VCAN* genes (3bINP-100), whereas in the three remaining patients, secondary findings were attributed to *RET*-, *TTN*-, and *MSH6*-related disorders (3bINP-045, 047, and 074, respectively). 

We documented three novel variants underlying these second autosomal dominant traits (Table 4). The variant NM_000168.6:c.3740_3743dup, or p.(Cys1249AlafsTer3), was found in *GLI3*, while the variant NM_000179.3:c.2150_2153del, or p.(Val717AlafsTer18), was documented in *MSH6*, and the variant NM_004385.5:c.3455C>A, or p.(Ser1152Ter), was found in *VCAN*. 

### 2.5. Syndromic Entities Not Related to IEiM Identified by WES

Two patients in Group 2 presented a nonspecific elevation of acylcarnitine and hyperbeta-alaninemia with a negative result in WES for IEiM but presented additional clinical abnormalities, suggesting syndromic entities responsible for intellectual disabilities not related to IEiM, including heterozygous pathogenic variants in *SOX4* and *PAFAH1B1* responsible for Coffin-Siris syndrome type 10 (3bINP-001) and lissencephaly type 1 (3bINP-041), respectively. 

### 2.6. Decisions Taken in Medical or Nutritional Management After WES Results

Based on WES results, in 32/95 cases (33.6%), a decision related to treatment had to be made, categorized as follows. (1) Modification of the initial treatment: in 18/32 patients (56.2%), the causes of these changes were discordance between the initial and final diagnosis, or the co-occurrence of a second monogenic trait or syndromic entities unrelated to IEiM, or initial unspecific diagnosis and a negative WES result. (2) Continuation of initial treatment: in 5/32 (15.6%) patients, despite their unsolved (inconclusive or negative) WES result, maintenance of the current treatment was decided due to clinical improvement. (3) No specific treatment was provided before or after WES because the initial unspecific biochemical findings were not confirmed in 9/32 (28.1%) patients (Table 5). 

## 3. Discussion

This study represents the first cohort of Mexican patients to assess the efficacy of WES in diagnosing IEiM. Overall, the WES diagnostic yield for both study groups was 72.6%, demonstrating that WES analysis facilitated the identification of the genotype responsible for IEiM diagnosis across our entire patient cohort. This study revealed a higher diagnostic yield than previously reported studies assessing IEM, i.e., 38.7% in Canadian patients (N = 12/31) [25] or 59% (N = 83/141) of Spanish newborns with a positive IEM screening result [26].

In Group 1, due to the previously well-defined biochemical phenotype, the concordance between the initial biochemical diagnosis and the final molecular diagnosis was remarkably high, reaching 91.30% (*N* = 63/69 patients), which contrasted with the substantially lower concordance rate (23.1%) documented in patients in Group 2 who presented unspecific metabolic alterations in amino acid and acylcarnitine profiles or other laboratory parameters (Figure 1). This demonstrates the fact that when a patient presents a specific biochemical profile, the probability of a positive WES result could be high. However, patients with unspecific biochemical profiles are even more in need of a WES test to discover or discard the presence of an IEiM or other genetic entity. A better diagnostic performance of NGS-based strategies supported on a previous detailed phenotypic delineation performed before genotyping in other monogenic traits has also been demonstrated for the heterogeneous group of mitochondrial diseases, i.e., a higher WES concordance was achieved in patients harboring a suggestive score for these disorders (49%, *N* = 29/59 patients) than in those lacking this previous clinical evaluation (28.8%, *N* = 17/59) [27]. This finding supports the importance of considering each patient’s previous complete clinical and biochemical assessment before performing WES analysis to improve the overall IEiM diagnostic yield. Additionally, as suggested by other groups [10,28], to increase the WES diagnostic yield in patients affected by a suspected monogenic disorder, all our included patients were first evaluated by clinical geneticists, in addition to the participation of molecular biologists, biochemists, bioinformaticians, nutritionists, and physicians highly trained in the diagnosis and management of inherited metabolic diseases. 

The higher concordance of WES reached in study Group 1 supports the notion that biochemical tests are still highly accurate tools for diagnosing IEiM [12], especially for urea cycle disorders, organic acidurias, and amino acid disorders, which showed a WES diagnostic concordance of 100%, 95.6%, and 90%, respectively (Figure 2B,E). Moreover, no discrepancies were observed between the initially assigned biochemical phenotype and the responsible genotype in the 63 patients in Group 1 (Figure 1 and Table 2). Therefore, our results suggest that biochemical tests should be performed immediately in patients with a suspected diagnosis of IEiM for prompt initiation of specific treatments to limit organ damage, especially brain sequelae [29]. Although NGS-based technologies can be time-consuming and expensive and their interpretation remains challenging in some cases [10], they have proven to be a reliable second-tier newborn screening (NBS) methodology by reducing false-positive results, facilitating the timely resolution of the case, and, in some cases, suggesting a more appropriate or specific diagnosis than that initially obtained by mass spectrometry [12]. 

We found that 6.3% (*N* = 6/95) of patients had inconclusive WES results. This percentage is lower than that reported by other authors in genetically heterogeneous diseases, such as neuromuscular disorders (21.9%, *N* = 9/41 [30]) and developmental epileptic encephalopathy (21.9%, *N* = 31/141 [31]), which could be related to the previously specific biochemical delineation available for most of our patients (*N* = 69/95; Figure 1).

Moreover, five patients in Group 1 presented inconclusive WES results, attributed to monoallelic pathogenic genotypes for *HLCS*- and *ACADM*-related disorders (*N* = 2/6, patients 3bINP-036 and 052) and biallelic genotypes for VUS *DBT*- and *GALK1*-related disorders (*N* = 3/6, patients 3bINP-076, 089, and 094), which could explain the previously biochemically diagnosed autosomal recessive IEiM (Table 3). Monoallelic *HLCS* genotypes seem to be infrequent findings in holocarboxylase synthetase deficiency (OMIM #253270), as reported recently in a small sample of Chinese patients biochemically confirmed with this IEiM, where Sanger sequencing revealed biallelic *HLCS* pathogenic genotypes in all the participants [32]. However, at least one affected patient with an apparent monoallelic *HCLS* genotype has been described along with a paracentric inversion of chromosome 21 disrupting the second *HLCS* allele [33]. Additionally, to the best of our knowledge, proven pathogenic deep intronic variants have not been described in the *HCLS* gene [34], although several gross deletions and duplications encompassing more than one exon have been described (ClinVar: https://www.ncbi.nlm.nih.gov/clinvar/?term=HLCS%5Bgene%5D&redir=gene, accessed on 20 May 2024). Moreover, our patient, 3bINP-036, received biotin at a dose of 20 mg per day, which improved his biochemical profile, which consisted of normalization of the hydroxy-pentanoylcarnitine (C5-OH) blood concentration and disappearance of the abnormal urinary organic acids (Table 3). However, since patient 3bINP-036 did not have the typical biochemical profile of holocarboxylase synthetase deficiency at diagnosis, which consists of elevated C5-OH, a urine organic acid profile with elevated lactic acid, 3-OH isovaleric, 3-OH propionic, 3-MCC, methylcitric acid, and tiglylglycine [35], further studies are warranted to confirm or discard this disease. 

Regarding the other monoallelic case with suspicion of medium-chain acyl-CoA dehydrogenase deficiency (MCADD, 3bINP-052), it is known that in European populations, such as Portuguese (77.9% of Gypsy origin) [36] and German [37] ones, Sanger sequencing identified biallelic diagnostic *ACADM* genotypes in 100% of analyzed patients; instead, the identification of monoallelic *ACADM* genotypes by traditional sequencing approaches seems to be common in Asian populations, as it has been identified in 8.7% (*N* = 2/23) of Chinese patients biochemically confirmed with MCADD [38] and in 14.3% of MCADD Japanese patients detected by NBS, even identifying normal *ACADM* genotypes [39]. Unfortunately, a reliable estimation of monoallelic *ACADM* genotypes in Latino-derived MCADD populations is lacking. As our patient, 3bINP-052, bearing the p.(Lys329Glu) *ACADM* allele (rs77931234), which has been identified in 80% of European-origin MCADD patients [40], had a typical biochemical MCADD acylcarnitine profile consisting of elevated levels of hexanoylcarnitine (C6), octanoylcarnitine (C8), and decanoylcarnitine (C12), further identification of a second pathogenic allele via other molecular approaches seems plausible. This patient was initially identified by an abnormal NBS result that revealed elevated blood concentrations of C6, C8, and C12, and was referred to our metabolic center to confirm those results. We found the same metabolic pattern suggestive of MCADD; thus, immediate treatment recommendations were started, consisting of frequent meals and avoidance of formulas with medium-chain triglycerides, along with strict medical follow-up in our clinic. At the time of this study, the patient was five years old and had no symptomatology or metabolic crisis associated with MCADD.

Searching for an eventual second pathogenic allele in these two previously described patients could be addressed in the future by applying long-read whole-genome sequencing or RNA-seq methodologies [41], as demonstrated across various monogenic conditions, including autosomal recessive metabolic disorders [42,43]. In particular, RNA-seq has been demonstrated to increase the diagnostic yield of these disorders by 10%–16% compared with WES alone [41]. 

Additionally, our patients with inconclusive or even negative WES results could be candidates for performing a later reanalysis of their WES data, as it has been estimated that this reassessment 1–3 years after the initial report may increase the diagnostic yield by 3–15% [41]. Additionally, for those genetic conditions in which a copy number variant (CNV) has been implicated, chromosomal microarray analysis can allow the identification of the other variant [41]. Unfortunately, this approach was unsuccessful in identifying the expected second pathogenic *HLCS* allele in our patient, 3bINP-036 (Table 3).

With respect to the three patients in Group 1 bearing inconclusive biallelic VUS genotypes, additional future strategies, such as in vitro or in vivo functional studies, may be warranted to reclassify these missense VUSs [41,44], considering the highly suggestive metabolic findings observed in these patients, or simply by awaiting the description of other affected patients bearing these same alleles. Moreover, due to the evident biochemical profile (elevated blood concentrations of branched-chain amino acids, including alloisoleucine) and the clinical phenotype highly suggestive of MSUD in patients 3bINP-076 and 094 (Table 3), along with the clinical improvement by specific medical and nutritional treatments observed in both cases and by considering the severity and potentially lethal nature of this disease, the medical decision was to maintain these treatments. The same criterion was applied to patient 3bINP-089 bearing two *GALK1* missense VUSs, which seems explain the high blood concentrations of galactose; thus, medical and nutritional treatments were sustained. 

Remarkably, the only inconclusive case in Group 2 (patient ID 3bINP-012; Table 3) was possibly related to the very uncommon phenomenon attributed to genome-wide paternal uniparental disomy identified in nearly 0.0002% of patients subjected to clinical exome or chromosomal microarray analyses [45]. This patient is still under study.

With respect to the negative WES results obtained in 20 patients (21.05%; Figure 1 and Table 3), lipid metabolism defects showed the lowest concordance since the genetic cause was demonstrated in only 33.3% (*N* = 2/6) of the patients. It has been estimated that when WES/WGS are applied as first-tier tests, negative results are common (42.5% to 66%) in most of the studied cohorts of patients [28,44]. It will be essential to consider applying further analysis to determine whether their isolated or persistent nonspecific biochemical abnormalities are due to undetected genetic defects, i.e., promoter or deep intronic variants, CNVs, epistatic–epigenetic mechanisms, low-grade mosaicisms, common pathogenic variants undetected by bioinformatic algorithms, or synergistic oligogenic heterozygosity [8,44], or whether these abnormalities are simply related to environmental factors (exposomes), such as malnutrition, energetic imbalances, prescribed drugs, or infections [1,41,46,47]. In these patients, applying complementary genetic and functional tests, such as trio-WES, WGS, RNA-Seq, epigenomics, metabolomics, proteomics, or optical genome mapping, could be considered in the future. These approaches are crucial for ruling out a genetic etiology for patients with highly suspected inherited disease and negative WES results [12,41,43,44,47]. Remarkably, a single patient in Group 1 had a negative WES result (3bINP-072; Figure 1 and Table 3). This patient was a 29-gestational-week preterm female weighing 1.2 kg at birth who immediately developed respiratory distress syndrome requiring mechanical ventilatory support. She later developed broncho dysplasia, necrotizing enterocolitis requiring ileostomy, retinopathy, and renal tubular acidosis. Biochemically, she presented with hypoglycemia, hyperammonemia, hyperlactatemia, metabolic acidosis, and elevated liver transaminases. An abdominal ultrasound revealed hepatomegaly but no parenchymal structural alterations, splenomegaly, or ascites. A liver biopsy revealed a significant glycogen load in hepatocytes compatible with the diagnosis of glycogen storage disease (GSD) type I, which was not genotypically confirmed by WES. Therefore, other diagnostic possibilities must be ruled out. The patient has been gradually released from the GSD diet. 

Concerning the characterized IEiM mutational spectrum described herein, we noted a high proportion of homozygous genotypes (50%), which could be related to the consanguinity (14.73%) and endogamy (23.15%) recorded in our patients. This finding is consistent with our previous report of IEiM families, in which 13.5% consanguinity was documented [23], but is lower than the worldwide rate observed in patients with IEiM (51%) [48]. This finding highlights the importance of providing genetic counseling for these families. 

Notably, we only documented the following three novel variants, as they have not been previously reported in public databases, such as the Leiden Open Variation Database v.3.0 (LOVD, https://www.lovd.nl/; accessed on 20 May 2024), dbSNP (https://www.ncbi.nlm.nih.gov/snp/; accessed on 20 May 2024), Genome Aggregation Database (https://gnomad.broadinstitute.org/; accessed on 20 May 2024), ClinVar (https://www.ncbi.nlm.nih.gov/clinvar/; accessed on 20 May 2024), and The Human Genome Mutation Database (https://www.hgmd.cf.ac.uk/; accessed on 20 May 2024), or to the best of our knowledge, in the literature: NM_000159.4(*GCDH*):c.1173_1174insT, or p.(Asn392Ter), NM_005271.5(*GLUD1*):c.1466C>G, or p.(Pro489Arg), and NM_001370658.1(*BTD*):c.1352G>C, or p.(Cys451Ser) (Table 2, Supplementary Table S1). The nonsense p.(Asn392Ter) variant in *GCDH* was considered pathogenic, whereas the missense variants in *GLUD1* p.(Pro489Arg) and *BTD* p.(Cys451Ser) were considered LP. 

In particular, the novel *GCDH* p.(Asn392Ter) variant was detected in *trans* with the pathogenic p.(Arg234Trp) variant in patient 3bINP-023. The p.(Arg234Trp) variant has been previously identified in homozygous state in two affected Polish sisters with a milder phenotype of glutaric acidemia, type I (OMIM #231670) [49]. In our patient, we observed a mild phenotype characterized by intellectual disability, seizures, abnormal movements, dyskinetic syndrome, truncal ataxia and dysmetria, abnormalities of the cerebral white matter and basal gray nuclei, along with malnutrition. 

The LP variant p.(Pro489Arg) in *GLUD1*, which is responsible for autosomal dominant hyperinsulinism–hyperammonemia syndrome (OMIM #606762), was confirmed by Sanger sequencing in the heterozygous state in both patient 3bINP-085 and his father. After the identification of the variant in the apparently healthy father, a blood ammonia concentration test, which had a value of 216 ng/dL (reference range 31-123 ng/dL), was requested. This male patient, 3bINP-085, also presented co-occurrence with X-linked hemolytic anemia due to G6PD deficiency (OMIM #300908), which was attributed to the most prevalent worldwide *G6PD* haplotype, c.[202G>A;376A>G], or p.[Val68Met;Asn126Asp], conditioning G6PD deficiency [50].

The third novel *BTD* variant, p.(Cys451Ser), was present in a homozygous state in a five-year-old male patient (3bINP-090; Table 2) affected by autosomal recessive biotinidase deficiency (OMIM #253260), whose parents reported inbreeding and consanguinity. This patient exhibited mild intellectual disability, poor visual acuity, nerve optic atrophy, and pectus excavatum. As the missense p.(Cys451Ser) variant was classified as LP, we performed direct genotyping via Sanger sequencing on the parents, confirming their obligate carrier status and subsequently supporting its pathogenicity.

The co-occurrence of two monogenic traits was documented in 10.5% (*N* = 10/95) of our studied patients. This is quite similar to that reported (10.4%) in most studies included in a recent systematic review [28], although great variability has been noted in other series (nearly 5%) [51,52]. Most of these secondary findings are related to cardiovascular disease and hereditary cancer syndromes [28]; however, in some instances, G6PD deficiency is the second most frequently identified monogenic trait [51], as it is considered the most common enzymopathy among humans, affecting over 500 million people worldwide [50]. In our study population, 40% (*N* = 4/10; Table 4) of the overall identified secondary monogenic disorders were distributed among the three major disease categories reported. 

The secondary actionable findings (i.e., cardiovascular disease or hereditary cancer syndromes) accounted for 3.15% (*N* = 3/95) of the overall study population (Table 4), which is in accordance with the 1-6% previously reported [10,53]. 

Finally, a treatment decision was taken after WES results in 24.2% (*N* = 23/95) of the studied patients. Changes in medical management were mainly related to the co-occurrence of a second monogenic disorder (*N* = 7/23). In fact, despite that in some patients the WES analysis results removed the suspicion of carrying an IEiM, it allowed the identification of other monogenic actionable disorders (i.e., patients 3bINP-001 and 3bINP-041), leading to a redirection of the medical management (Table 5). Changes in medical or nutritional management can be of different types, including redirection of care, initiation of new subspecialist care, changes in diet or medication, or major procedures, such as liver or kidney transplant [54]. In general, it is estimated that therapy guided by NGS results can reach 14.6% of the analyzed patients [28], although these proportions differ significantly among different studies, reaching 45.5% (*N* = 10/22) [9] to 52% in critically ill studied patients after WES analysis [10,54]. Importantly, the cohorts studied by the formerly mentioned authors were mainly composed of severely ill patients. In contrast, our cohort was entirely composed of metabolically compensated ambulatory patients who were previously diagnosed and treated according to their biochemical profile. 

## 4. Materials and Methods

### 4.1. Study Population 

An observational, descriptive, prospective, and cross-sectional study was conducted. Patients and their parents were contacted by telephone and invited to participate. The study included 95 unrelated Mexican mestizo individuals (51 females and 44 males) recruited from a cohort of pediatric patients (mean age 9 years) who attended the National Institute of Pediatrics, Mexico (https://www.pediatria.gob.mx/; accessed on 27 October 2024). Demographic data were registered. Patients with methylmalonic/propionic acidemia or hyperphenylalaninemia were not included in this study, as they are included in other institutional protocols and/or their mutational spectrum has already been reported [23,55].

Patients were categorized into one of the following classes based on their biochemical phenotypes: amino acid, urea cycle, organic acid, carbohydrate, or lipid disorders. The included patients were assigned to two groups. Those bearing a well-defined biochemical phenotype that indicated a specific IEiM were assigned to Group 1, i.e., a high blood concentration of arginine was indicative of argininemia [56,57]. Group 2 included patients with either persistent or isolated nonspecific alterations in their amino acid and acylcarnitine profiles or with unexplained abnormalities in other laboratory studies, such as hypoglycemia and hyperammonemia. 

### 4.2. Biochemical Testing and Phenotyping

In all cases, dried blood spot (DBS) samples, obtained from heel prick (in patients under 6 months of age) or finger prick (in patients above 6 months of age) via a standard protocol, were used to quantify amino acids, acylcarnitines, and succinylacetone quantification via tandem mass spectrometry using a conventional methodology previously described [58]. A plasma sample was also obtained for amino acid quantification via high-performance liquid chromatography (HPLC), and urinary organic acids were analyzed via gas chromatography coupled with mass spectrometry (GC/MS). In some cases, orotic acid was determined from urine. These determinations were made according to previously reported methodologies [58].

### 4.3. WES and Variant Analysis

Genomic DNA was extracted from DBS samples via the standard salting-out method. WES was performed via the xGen Exome Research Panel v2, either by itself or supplemented with the xGen human mtDNA panel and the xGen Custom Hyb Panel v1 (Integrated DNA Technologies, Coralville, Iowa, USA), and the Illumina NovaSeq 6000 platform (Illumina, San Diego, CA, USA) was used to capture and sequence the protein-coding exons of ~20,000 known genes. The sequencing data were aligned to the GRCh37/hg19 human reference genome and the mitochondrial genome’s Revised Cambridge Reference Sequence (rCRS). Single-nucleotide variants (SNVs), small insertions and deletions (INDELs), copy number variants spanning at least 3 consecutive exons (CNVs), repeat expansion variants, and regions of homozygosity were called with open-source bioinformatics tools and in-house software, as previously described, and this was performed for positive, negative, and inconclusive patients [59]. Variant annotation, filtering, and classification were performed via EVIDENCE [59]. Common variants with allele frequencies > 5% in the gnomAD database [60] or >1% internally were filtered out, except for known pathogenic/likely pathogenic (P/LP) variants. Variant classification was performed based on the American College of Medical Genetics and Genomics (ACMG) and the Association for Molecular Pathology (AMP) guidelines [16], along with the quantitative Bayesian scoring system [61]. All patients were subjected to clinical genetics assessment (A. G.-A., L. F.-H., and B. E.-O.) before their inclusion in this study. The patients’ clinical signs and symptoms were registered according to the Human Phenotype Ontology (HPO, https://hpo.jax.org/; accessed on 20 February 2024) [62], such that the symptom similarity score could be calculated [63,64] between the patient’s phenotype and that expected for ~7000 rare genetic diseases. Only variants deemed clinically significant and relevant to the patient’s primary clinical indications at the time of variant interpretation were reported. The clinical geneticists manually evaluated all these variants, prioritized by the classification and symptom similarity score to select the most likely genotype responsible for the diagnosed or suspected monogenic disease. 

Patients were classified as positive when their genotype involved “pathogenic” or “likely pathogenic” variant(s) correlating with their phenotype and its inheritance mode. In contrast, inconclusive classification was determined when two variants of uncertain clinical significance (VUS) were identified or when only one P/LP variant partially explained a suspected or biochemically diagnosed autosomal recessive IEiM. Patients without any identifiable IEiM-associated variants were classified as negative. Co-occurrence was defined as the presence of a second monogenic entity in the same patient, including those expected findings according to the recorded clinical and biochemical phenotypes, and incidental or secondary findings. According to the ACMG, incidental findings are defined as results that are not related to the indication for ordering the sequencing but that may nonetheless be of medical value or utility [65,66]. On the other hand, secondary findings are defined as known pathogenic or expected pathogenic variants in a defined set of genes considered medically actionable, even when unrelated to the primary medical reason for testing [67]. Incidental and secondary findings were reported following the ACMG SF v.3.2. list [67]. Directed Sanger sequencing was performed on selected variants in 14 patients and, when available, on their parents to attempt to reclassify VUS as a “LP” or “P” variant by demonstrating the *trans* configuration, clarifying the mode of inheritance, or to identify clinically relevant parental genotypes for genetic counseling purposes. Based on this, genotypes were indicated according to the HGVS guidelines version 21.0.4 (https://hgvs-nomenclature.org/stable/; accessed on 12 August 2024). 

### 4.4. Statistical Analyses

Differences in the concordance between the biochemical initial diagnosis and WES results were investigated by the two-sided Fisher’s exact test in the general cohort, as well as between the two studied groups and between types of disorders. A *p*-value < 0.05 was considered to be statistically significant (* *p* < 0.05, ** *p* < 0.01, *** *p* < 0.001, and **** *p* < 0.0001). If applicable, descriptive statistics were applied. These statistical determinations were performed with GraphPad Prism version 10.1.1 (GraphPad Software, Boston, MA, USA). 

### 4.5. Ethical Considerations

The Research, Ethics, and Biosafety Institutional Committees approved the protocol (institutional number 2022/051). Before carrying out the molecular study, the parents of the included patients signed an informed consent form, and those patients who could, due to their age and competence, granted their consent. Everyone was asked to decide whether they wanted to know about the secondary findings (ACMG SF v.3.2.) [67]. All the families received pre- and post-WES genetic counseling and medical follow-up. 

## 5. Conclusions

In the 95 studied unrelated Mexican pediatric patients included in this study, the previous specific biochemical diagnosis of an IEiM correlated with a higher genetic concordance of WES (91.3%, *N* = 63/69 patients) compared with unspecific biochemical alterations suggestive of these disorders (23.1%, *N* = 6/26 patients). The overall diagnostic concordance between the initial biochemical profile indicating an IEiM diagnosis and the responsible genotype identified through WES was 72.6% (*N* = 69/95 patients). These results highlight the importance of biochemical studies as a first-tier diagnostic approach in all patients with suspected IiEM to achieve prompt and specific implementation of therapeutic management, as well as to increase the overall diagnostic yield of WES. The identified underlying genotypic IEiM spectrum involved 83 pathogenic, likely pathogenic, and VUS variants, including three novel ones in *GLUD1*, *BTD,* and *GCDH*, which were distributed among 29 different genes responsible for amino acid, organic acid, urea cycle, carbohydrate, and lipid disorders. Unsolved WES results were identified in 27.4% (*N* = 26/95) of the patients. The proportion of patients with a second monogenic disease (10.5%) was similar to that reported in the literature (10.4%). The second monogenic diseases found were mainly cardiovascular, hereditary cancer syndromes, and G6PD deficiency. WES-directed modifications in medical or nutritional management were performed in 33.6% (*N* = 32/95) of patients. In 56.2% of them (*N* = 18/32), the changes were attributed to discordance between the initial and final diagnosis, the co-occurrence of a second monogenic trait or syndromic entities unrelated to IEiM, or an initial unspecific diagnosis with a negative WES result.

## Figures and Tables

**Table 1:** Gene variants responsible for IEiM identified in Mexican patients, categorized by pathogenicity.

Type of Disorder	Disease	Gene	Total Number of Variants	Pathogenic	Likely Pathogenic	Variant of Uncertain Significance
**Amino acid disorders**	Maple syrup urine disease (MSUD) type Ib (OMIM #620698)	***BCKDHB***	6	4	2	0
	MSUD, type II (OMIM #620699)	***DBT***	6	3	1	2
	MSUD, type Ia (OMIM #248600)	***BCKDHA***	2	1	1	0
	Homocystinuria, B6-responsive and nonresponsive types (OMIM #236200)	***CBS***	2	2	0	0
	Cystinosis, nephropathic (OMIM #219800)	***CTNS***	2	0	2	0
	Hyperornithinemia-hyperammonemia-homocitrullinuria syndrome (OMIM #238970)	***SLC25A15***	1	1	0	0
Organic acid disorders	HMG-CoA lyase deficiency, 3-OH-3-methylglutaric acidemia (OMIM #246450)	***HMGCL***	7	5	2	0
	Glutaric Acidemia Type 1 (OMIM #231670)	***GCDH***	5	5	0	0
	Isovaleric acidemia (OMIM #243500)	***IVD***	5	4	1	0
	Biotinidase deficiency (OMIM #253260)	***BTD***	4	3	1	0
	Mitochondrial DNA depletion syndrome 9 (encephalomyopathic type with methylmalonic aciduria) (OMIM #245400)	***SUCLG1***	2	1	1	0
	Beta-ketothiolase deficiency or mitochondrial acetoacetyl-CoA thiolase deficiency or alphamethylacetoacetic aciduria (OMIM #203750)	***ACAT1***	3	1	2	0
	Holocarboxylase synthetase deficiency (OMIM #253270)	***HLCS***	1	1	0	0
	3-Methylcrotonyl-CoA carboxylase 2 deficiency (OMIM #210210)	***MCCC2***	1	0	1	0
	Succinyl CoA:3-oxoacid CoA transferase deficiency (OMIM #245050)	***OXCT1***	1	1	0	0
Urea cycle disorders	Argininemia (OMIM #207800)	***ARG1***	8	5	3	0
	Citrullinemia (OMIM #215700)	***ASS1***	6	4	2	0
	Ornithine transcarbamylase deficiency (OMIM #311250)	***OTC***	2	2	0	0
	Argininosuccinic aciduria (OMIM #207900)	***ASL***	1	0	1	0
Carbohydrate disorders	Glycogen storage disease Ia (OMIM #232200)	***G6PC1***	5	4	1	0
	Glycogen storage disease IIIb (OMIM #232400)	***AGL***	1	0	1	0
	Glycogen storage disease Ib (OMIM: 232220)	***SLC37A4***	2	1	1	0
	Diarrhea type 4, malabsorptive, congenital (OMIM #610370)	***NEUROG3***	1	1	0	0
	Galactokinase deficiency with cataracts (OMIM #230200)	***GALK1***	2	0	0	2
	Hyperinsulinemic hypoglycemia, familial, type 1 (OMIM #256450)	*** ABCC8 ***	1	1	0	0
	Hyperinsulinism-hyperammonemia syndrome (OMIM #606762)	***GLUD1***	1	0	1	0
	Hyperinsulinemic hypoglycemia, familial, type 2 (OMIM #601820)	***KCNJ11***	1	0	1	0
Lipid disorders	Acyl-CoA dehydrogenase, medium chain deficiency (MCAD, OMIM #201450)	***ACADM***	3	3	0	0
	Hypercholesterolemia familial type 1 (OMIM #143890)	***LDLR***	1	1	0	0
		Total	83	54	25	4

**Table 2:** Genotypic spectrum underlying IEiM in positive cases of the studied population.

Amino Acid Disorders								
Biochemical Phenotype	Initialy Suspected Disease	Responsible Gene (Reference Sequence)	Patient ID	LOVD Individual Accession Number	Genotype ^A^	Protein Change	Final Diagnosis	Inheritance
Elevated circulating branched chain amino acid concentration (HP:0008344)	MSUD	*BCKDHB* (*NM_183050.4*)	3bINP-066	451632	c.[152del];[152del]	p.[Val51GlyfsTer21];[Val51GlyfsTer21]	MSUD type Ib (OMIM #620698)	AR
			3bINP-080	451644	c.[564T>A];[564T>A]	p.[Cys188Ter];[Cys188Ter]		
			3bINP-020	451365	c.564T>A(;)1087T>A	p.(Cys188Ter)(;)(Tyr363Asn)		
			3bINP-077	451640	c.853C>T(;)667G>C	p.(Arg285Ter)(;)(Gly223Arg)		
			3bINP-004	450321	c.[970C>T];[970C>T]	p.[Arg324Ter];[Arg324Ter]		
			3bINP-013	450471	c.[1087T>A];[1087T>A]	p.[Tyr363Asn];[Tyr363Asn]		
		*DBT* (*NM_001918.5*)	3bINP-069	451637	c.[75_76del];[75_76del] ^B^	p.[Cys26TrpfsTer2];[Cys26TrpfsTer2]	MSUD type II (OMIM #620699)	
			3bINP-092	451652				
			3bINP-104	451662	c.[263_265del];[263_265del]	p.[Glu88del];[Glu88del]		
			3bINP-027	451439	c.[434-15_434-4del];[434-15_434-4del]	p.[?];[?]		
			3bINP-081	451645	c.670G>T(;)434-15_434-4del	p.(Glu224Ter)(;)(?)		
		*BCKDHA* (*NM_000709.4*)	3bINP-062	451630	c.890G>A(;)1192G>T	p.(Arg297His)(;)(Glu398Ter)	MSUD type Ia (OMIM #248600)	
Hyperammonemia (HP:0001987)	Gyrate atrophy or HHH Sx	*SLC25A15* (*NM_014252.4*)	3bINP-021	451367	c.[113_116dup];[113_116dup]	p.[Phe40AspfsTer4];[Phe40AspfsTer4]	Hyperornithinemia-hyperammonemia-homocitrullinuria syndrome (OMIM #238970)	AR
Homocystinuria (HP:0002156)	Homocystinuria	*CBS* (NM_000071.3)	3bINP-046	451595	c.[572C>T];[572C>T] ^C^	p.[Thr191Met];[Thr191Met]	Homocystinuria (OMIM #236200)	AR
			3bINP-087	451647				
			3bINP-109	451663				
			3bINP-049	451597	c.[1126G>A];[1126G>A]	p.[Asp376Asn];[Asp376Asn]		
Corneal crystals (HP: 0000531), Fanconi syndrome (HP: 0011463) ^C^	Cystinosis	*CTNS* (*NM_004937.3*)	3bINP-082	451648	c.[22_23del];[1036_1047del] ^D^	p.[Ile8PhefsTer13];[Asp346_Phe349del]	Cystinosis, nephropathic (OMIM #219800)	AR
**Organic acid disorders**								
**Biochemical phenotype**	**Initialy suspected disease**	**Responsible gene**	**Patient ID**	**LOVD individual accession number**	**Genotype ^A^**	**Protein change**	**Final diagnosis**	**Inheritance**
Decreased circulating biotinidase concentration (HP:0410145)	Biotinidase deficiency	*BTD* (*NM_001370658.1*)	3bINP-054	451617	c.468G>T(;)1270G>C	p.(Lys156Asn)(;)(Asp424His)	Biotinidase deficiency(OMIM #253260)	AR
	Biotinidase deficiency with atypical outcome, epilepsy		3bINP-101	451656	c.[754T>G];[754T>G]	p.[Trp252Gly];[Trp252Gly]		
	Biotinidase deficiency		3bINP-053	451616	c.1270G>C(;)754T>G	p.(Asp424His)(;)(Trp252Gly)		
	Biotinidase deficiency		3bINP-011	450470	c.[1270G>C];[1270G>C]	p.[Asp424His];[Asp424His]		
Elevated circulating acylcarnitine concentration (HP: 0045045), organic aciduria (HP:0001992)	Multiple carboxylase deficiency		3bINP-090	451651	**c.[1352G>C];[1352G>C]**	**p.[Cys451Ser];[Cys451Ser]**		
Organic aciduria (HP:0001992)	Multiple carboxylase deficiency	*SUCLG1* (*NM_003849.4*)	3bINP-028	451440	c.40A>T(;)548T>C	p.(Met14Leu)(;)(Ile183Thr)	Succinate-CoA ligase, alpha subunit deficiency (OMIM #245400)	AR
Elevated circulating acylcarnitine concentration (HP:0045045)	Multiple carboxylase deficiency		3bINP-084	451646				
Increased circulating isovaleric acid concentration (HP:0033148)	Isovaleric acidemia	*IVD* (*NM_002225.5,* *NC_000015.9*)	3bINP-057	451600	c.[149G>C];[149G>C]	p.[Arg50Pro];[Arg50Pro]	Isovaleric acidemia (OMIM #243500)	AR
			3bINP-019	451363	c.[850C>T];[850C>T]	p.[Arg284Trp];[Arg284Trp]		
			3bINP-003	450320	c.[1065G>C];[1065G>C]	p.[Lys355Asn];[Lys355Asn]		
			3bINP-079	451643	c.[1175G>A];[1175G>A]	p.[Arg392His];[Arg392His]		
			3bINP-005	450322	g.[(?_40710329)_(40710462_?)del];[(?_40710329)_(40710462_?)del](homozygous exon 12 deletion)	p.[?];[?]		
3-methylglutaric aciduria (HP:0410051)	3-hydroxy-3-methylglutaryl-CoA lyase deficiency	*HMGCL* (*NM_000191.3*)	3bINP-042	451460	c.[109G>T];[109G>T]	p.[Glu37Ter];[Glu37Ter]	3-hydroxy-3-methylglutaryl-CoA lyase deficiency (OMIM #246450)	AR
			3bINP-035	451457	c.[112G>T];[112G>T]	p.[Val38Phe];[Val38Phe]		
			3bINP-010	450325	c.121C>T(;)233C>T	p.(Arg41Ter)(;)(Ser78Phe)		
			3bINP-074	451639	c.230del(;)31C>T	p.(Val77GlyfsTer16)(;)(Arg11Ter)		
			3bINP-032	451445	c.[505_506del];[505_506del]	p.[Ser169LeufsTer8];[Ser169LeufsTer8]		
Concentration of glutaric acid in the urine above the upper limit of normal (HP:0003150)	Glutaric aciduria	*GCDH* (*NM_000159.4*)	3bINP-018	451362	c.263G>A(;)1204C>T	p.(Arg88His)(;)(Arg402Trp)	Glutaric aciduria (OMIM #231670)	AR
			3bINP-023	451436	c.[700C>T];**[1173_1174insT]** ^**G**^	p.[Arg234Trp];**[Asn392Ter]**		
			3bINP-068	451635	c.[1082+31_1243+678del];[1082+31_1243+678del](homozygous whole exon 11 deletion)	p.[Ala362Tyrfs*3];[Ala362TyrfsTer3]		
Elevated circulating acylcarnitine concentration (HP:0045045)	Organic aciduria	*ACAT1* (*NM_000019.4*)	3bINP-047	451615	c.[473A>G];[473A>G]	p.[Asn158Ser];[Asn158Ser]	Alpha-methylacetoacetic aciduria (OMIM #203750)	AR
Organic aciduria (HP:0001992)	Organic aciduria		3bINP-017	450485	c.826+3_826+6del(;)200T>G	p.(?)(;)(Leu67Arg)		
Organic aciduria (HP:0001992)	3-Methylcrotonyl-CoA carboxylase deficiency	*MCCC2* (*NM_022132.5*)	3bINP-059	451602	c.[1356G>A];[1356G>A]	p.[Met452Ile];[Met452Ile]	3-Methylcrotonyl-CoA carboxylase deficiency (OMIM #210210)	AR
Ketosis (HP:0001946)	Ketone bodies defect	*OXCT1* (*NM_000436.4*)	3bINP-060	451603	c.[1243del];[1243del]	p.[Ile415TyrfsTer6];[Ile415TyrfsTer6]	Succinyl-CoA:3-oxoacid- CoA transferase deficiency (OMIM #245050)	AR
**Urea cycle disorders**								
**Biochemical phenotype**	**Initialy suspected disease**	**Responsible gene**	**Patient ID**	**LOVD individual accession number**	**Genotype ^A^**	**Protein change**	**Final diagnosis**	**Inheritance**
Hyperargininemia (HP:0500153)	Argininemia	*ARG1* (*NM_000045.4*)	3bINP-033	451456	c.3G>A(;)767_769del	p.(Met1?)(;)(Glu256del)	Argininemia (OMIM #207800)	AR
			3bINP-067	451634	c.61C>T(;)466-1G>C ^E^	p.(Arg21Ter)(;)(?)		
			3bINP-078	451642				
			3bINP-037	451458	c.61C>T(;)892G>C	p.(Arg21Ter)(;)(Ala298Pro)		
			3bINP-055	451599	c.[425G>A];[425G>A]	p.[Gly142Glu];[Gly142Glu]		
			3bINP-073	451638	c.466-1G>C(;)787G>T	p.(?)(;)(Glu263Ter)		
			3bINP-105	451658	c.425G>A(;)871C>T	p.(Gly142Glu)(;)(Arg291Ter)		
Elevated plasma citrulline (HP:0011966)	Citrullinemia	*ASS1* (*NM_054012.4*)	3bINP-022	451368	c.[34A>G];[34A>G]	p.[Ser12Gly];[Ser12Gly]	Citrullinemia (OMIM #215700)	AR
			3bINP-106	451659	c.[256C>T];[256C>T]	p.[Arg86Cys];[Arg86Cys]		
			3bINP-008	450323	c.256C>T(;)836G>A ^F^	p.(Arg86Cys)(;)(Arg279GIn)		
			3bINP-065	451631				
			3bINP-095	451654	c.256C>T(;)1194-19_1197dup	p.(Arg86Cys)(;)(?)		
			3bINP-015	450482	c.970G>A(;)40G>A	p.Gly324Ser)(;)(Gly14Ser)		
Orotic aciduria (HP:0003218)	Ornithine transcarbamylase deficiency	*OTC* (*NM_000531.6*)	3bINP-043	451463	c.[583G>A];[583=] (heterozygous female)	p.[Gly195Arg];[Gly=] (heterozygous female)	Ornithine transcarbamylase deficiency (OMIM #311250)	X-linked
			3bINP-048	451596	c.[803T>C];[0] (hemizygous male)	p.[Met268Thr];[0] (hemizygous male)		
Argininosuccinic aciduria (HP:0025630)	Argininosuccinic aciduria	*ASL* (*NM_000048.4*)	3bINP-014	450472	c.[209T>C];[209T>C]	p.[Val70Ala];[Val70Ala]	Argininosuccinic aciduria (OMIM #207900)	AR
**Carbohydrate disorders**								
**Biochemical phenotype**	**Initialy suspected disease**	**Responsible gene**	**Patient ID**	**LOVD individual accession number**	**Genotype ^A^**	**Protein change**	**Final diagnosis**	**Inheritance**
Abnormal hepatic glycogen storage (HP:0500030), hypoglycemia (HP:0001943)	Glycogen storage disease type I	*G6PC1* (*NM_000151.4*)	3bINP-029	451441	c.379_380dup(;)1039C>T	p.(Tyr128ThrfsTer3)(;)(Gln347Ter)	Glycogen storage disease Ia (OMIM #232200)	AR
Hepatomegaly (HP:0002240), hypoglycemia (HP:0001943), hypertriglyceridemia (HP:0002155), hypercholesterolemia (HP:0003124), hepatic steatosis (HP:0001397), abnormal hepatic glycogen storage (HP:0500030)			3bINP-107	451660	c.[533C>T];[500G>A]	p.[Pro178Leu];[Cys167Tyr]		
Hepatomegaly (HP:0002240), hypoglycemia (HP:0001943)			3bINP-102	451657	c.[809G>T];[809G>T]	p.[Gly270Val];[Gly270Val]		
Abnormal hepatic glycogen storage (HP:0500030), hypoglycemia (HP:0001943)	Glycogen storage disease type III	*AGL* (*NM_000642.3*)	3bINP-025	451437	c.[2803G>T];[2803G>T]	p.[Gly935Cys];[Gly935Cys]	Glycogen storage disease IIIb (OMIM #232400)	AR
Abnormal hepatic glycogen storage (HP:0500030), hypoglycemia (HP:0001943), neutropenia (HP: 0001875)	Glycogen storage disease Ib	*SLC37A4* (*NM_001164277.1*)	3bINP-097	451655	c.82C>T(;)1130G>A	p.(Arg28Cys)(;)(Gly377Asp)	Glycogen storage disease Ib (OMIM #232220)	AR
Type 1 diabetes mellitus (HP:0100651)	Mauriac syndrome, type 1 diabetes mellitus	*NEUROG3* (*NM_020999.4*)	3bINP-100	451661	c.[117del];[117del]	p.[Thr40LeufsTer38];[Thr40LeufsTer38]	Diarrhea type 4, malabsorptive, congenital (OMIM #610370)	AR
Hypoglycemia (HP:0001943)	Hypoglycemia	*ABCC8* (*NM_000352.6*)	3bINP-045	451461	c.[2506C>T];[2506=] ^H^	p.[Arg836Ter];[Arg=]	Hyperinsulinemic hypoglycemia, familial, type 1 (OMIM #256450)	AD
Hypoglycemia, abnormal circulating glucose-6-phosphate dehydrogenase concentration (HP:0001943, HP:0410176)	Hypoglycemia and glucose-6-phosphate dehydrogenase deficiency	*GLUD1* (*NM_005271.5*)	3bINP-085	451650	**c.[1466C>G];[1466=]**	**p.[Pro489Arg];[Pro=]**	Hyperinsulinism-hyperammonemia syndrome (OMIM #606762)	AD
Hypoglycemia (HP:0001943)	Hypoglycemia	*KCNJ11* (*NM_000525.4*)	3bINP-030	451442	c.[560C>T];[560=] ^H^	p.[Ala187Val];[Ala=]	Hyperinsulinemic hypoglycemia, familial, type 2 (OMIM #601820)	AD
**Lipid defects**								
**Biochemical phenotype**	**Initialy suspected disease**	**Responsible gene**	**Patient ID**	**LOVD individual accession number**	**Genotype ^A^**	**Protein change**	**Final diagnosis**	**Inheritance**
Elevated circulating acylcarnitine concentration (HP:0045045)	Acyl-CoA dehydrogenase deficiency (MCAD)	*ACADM* (*NM_000016.6*)	3bINP-044	451464	c.799G>A(;)959C>A	p.(Gly267Arg)(;)(Ser320Ter)	MCAD (OMIM #201450)	AR
Hypercholesterolemia (HP:0003124)	Familial hypercholesterolemia	*LDLR* (*NM_000527.5*)	3bINP-058	451601	c.[337dup];[337=]	p.[Glu113GlyfsTer17];[Glu=]	Familial hypercholesterolemia type 1 (OMIM #143890)	AD

^A^ Described genotypes considered only pathogenic and likely pathogenic variants (see Supplementary Table S1). ^B, E, F^ Two patients sharing the same indicated genotype. ^C^ Three patients sharing the same indicated genotype. ^D^ A second monogenic disease was found in this patient, showing rapid progressive renal failure despite early pharmacological treatment with cysteamine (see Table 4). ^G^ Allele c.1173_1174insT was initially not detected by WES due to low-coverage issues, but it was further identified by the whole-genome sequencing approach focused on the *GCDH* sequence. ^H^ An autosomal dominant mode of inheritance was supported by demonstrating an affected phenotype in the heterozygous father and a normal homozygous genotype in the healthy mother. Novel variants are highlighted in bold. Genotypes are presented by ascendent nucleotide numbers. LOVD: Leiden Open Variation Database v.3.0.

**Table 3:** Biochemical primary biomarkers related to unsolved cases (inconclusive and negative) in the studied population.

Study Group	Patient ID	HPO	Biomarker	Concentration (Reference Value)	Suspected Disease	Gene (Reference Sequence, Encoded Protein)	Variant 1 (Classification)	Variant 2 (Classification)	Conclusion
1	3bINP-036	0045045	3-hydroxy-isovalerylcanitine + methylmalonylcarnitine	1.36 μmol/L (0.83)	Organic acidemia	*HLCS* (NM_001352514.2, Holocarboxylase synthetase)	c.2361_2362insT or p.(Val788CysfsTer108) (pathogenic)	Not identified	Inconclusive ^A^
	3bINP-052	0045045	Hexanoylcarnitine	0.16 μmol/L (0.12)	Medium chain acyl-CoA dehydrogenase deficiency	*ACADM* (NM_000016.6, Medium chain acyl-CoA dehydrogenase)	c.985A>G or p.(Lys329Glu) (pathogenic)	Not identified	Inconclusive ^B^
			Octanoylcarnitine	0.36 μmol/L (0.16)					
			Decanoylcarnitine	0.37 μmol/L (0.21)					
	3bINP-072	0500030	Glycogen	Positive liver biopsy	Glycogen storage disease	Not identified	Not identified	Not identified	Negative
	3bINP-076	0008344	Leucine + isoleucine	1924 μmol/L (40–228)	Maple syrup urine disease	*DBT* (NM_001918.5, Dihydrolipoamide branched-chain transacylase)	c.1210-3T>A or p.(?) (VUS)	c.1210-3T>A or p.(?) (VUS)	Inconclusive
			Valine	443 μmol/L (37–237)					
			Alloisoleucine	Not determined					
		0001992	Urinay organic acid profile	Elevated excretion of branched chain keto acids					
	3bINP-089	0012024	Galactose (with Galactose-1-P uridyltransferase normal activity)	19.99 mg/dL (<12)	Galactosemia	*GALK1* (NM_000154.2, Galctose kinase)	c.56C>A or p.(Ala19Asp) (VUS)	c.182C>T or p.(Thr61Met) (VUS)	Inconclusive
	3bINP-094	0008344	Leucine + isoleucine	3226 μmol/L (<253)	Maple syrup urine disease	*DBT* (NM_001918.5, Dihydrolipoamide branched-chain transacylase)	c.1261G>T or p.(Gly421Trp) (VUS)	c.1261G>T or p.(Gly421Trp) (VUS)	Inconclusive
			Valine	1286 μmol/L (<282)					
			Alloisoleucine	64 μmol/L (Not detectable)					
		0001992	Urinay organic acid profile	Elevated excretion of branched chain keto acids					
2	3bINP-001	0004359	Propionylcarnitine	4.6 μmol/L (<2.5)	Organic acidemia	Not identified	Not identified	Not identified	Negative
	3bINP-006	0045045	Tetradecanoylcarnitine	0.33 μmol/L (<0.31)	Organic acidemia	Not identified	Not identified	Not identified	Negative
		0001992	Urinay organic acid profile	Elevated excretion of adipic, suberic and sebasic acids					
	3bINP-007	0008358	Proline	393 μmol/L (<290)	Hyperprolinemia	Not identified	Not identified	Not identified	Negative
	3bINP-009	0001943	Glucose	<40 mg/dL (70)	Carbohydrate disorder	Not identified	Not identified	Not identified	Negative
		0000842	Hyperinsulinemia	19.3 uU/mL (<2)					
	3bINP-012	0001943	Glucose	<40 mg/dL (70)	Carbohydrate disorder	Not identified	Not identified	Not identified	Inconclusive ^C^
		0000842	Hyperinsulinemia	26.8 uU/mL (<2)					
	3bINP-016	0003235	Methionine	99 μmol/L (9–42)	Hypermethioninemia	Not identified	Not identified	Not identified	Negative
		0002156	Homocysteine	8 μmol/L (0–6.4)					
	3bINP-024	0001987	Hyperammonemia	117 μmol/L (9–35)	Urea cycle disorder	Not identified	Not identified	Not identified	Negative
	3bINP-026	0003348	Alanine	1007 μmol/L (<605)	Hyperalaninemia	Not identified	Not identified	Not identified	Negative
	3bINP-031	0003235	Methionine	209 μmol/L (<52),	Hypermethioninemia	Not identified	Not identified	Not identified	Negative
			Met/Phe ratio	Met/Phe 4.2 (<1.4)					
	3bINP-034	0045045	Hexadecanoylcarnitine	2.98 μmol/L (<2.4)	Fatty acid oxidation defect	Not identified	Not identified	Not identified	Negative
			Octadecenoylcarnitine	2.45 μmol/L (<1.6)					
			3-hydroxy-octadecenoylcarnitine	0.06 μmol/L (<0.03)					
			Octadecadienoylcarnitine	0.69 μmol/L (<0.47)					
	3bINP-038	0008344	Leucine + isoleucine	426 (<253)	Maple syrup urine disease	Not identified	Not identified	Not identified	Negative
			Valine	446 μmol/L (<282)					
			Xleu (Leu + Ile)/Phe ratio	7.12 (<3.95)					
			Xleu (Leu + Ile)/Ala ratio	10.7 (<0.43)					
			Val/Phe ratio	7.42 (<4.95)					
	3bINP-039	0008358	Proline	338 μmol/L (<290)	Hyperprolinemia	Not identified	Not identified	Not identified	Negative
	3bINP-041	0012556	beta-Alanine	8 μmol/L (<5)	Hyperbeta-alaninemia	Not identified	Not identified	Not identified	Negative
		0020079	beta-Alaninuria	256 mmol/mol creatinine (<6)					
		0500138	Serine	194 μmol/L (85-185)					
		0002154	Glycine	356 μmol/L (138-349)					
	3bINP-050	0001992	Propionylcarnitine	10.7 μmol/L (<4.3)	Organic acidemia	Not identified	Not identified	Not identified	Negative
	3bINP-051	0045045	Free carnitine	134 μmol/L (<53)	Fatty acid oxidation defect	Not identified	Not identified	Not identified	Negative
			Propionylcarnitine	29 μmol/L (<4.2)					
			Butyrylcarnitine	1.5 μmol/L (<0.5)					
			Hexadecanoylcarnitine	5.1 μmol/L (<2.2)					
			Tetradecanoylcarnitine	0.41 μmol/L (<0.19)					
			Octadecanoylcarnitine	2.7 μmol/L (<0.87)					
			Octadecenoylcarnitine	6.5 μmol/L (<2.8)					
	3bINP-056	0045045	Free carnitine	456 μmol/L (<87)	Fatty acid oxidation defect	Not identified	Not identified	Not identified	Negative
			Hexadecanoylcarnitine	0.32 μmol/L (<0.23)					
			Octadecanoylcarnitine	0.13 μmol/L (<0.1)					
			Free carnitine/(hexadecanoylcarnitine + octadecanoylcarnitine) ratio	1013 (<69)					
	3bINP-063	0001992	Urinay organic acid profile	Elevated excretion of 3-hydroxybutiric and acetoacetic acids	Organic acidemia	Not identified	Not identified	Not identified	Negative
	3bINP-070	0008344	Leucine + isoleucine	265 μmol/L (<253)	Maple syrup urine disease	Not identified	Not identified	Not identified	Negative
			Valine	303 μmol/L (<282)					
		0008358	Proline	439 μmol/L (<290)					
	3bINP-093	0008344	Leucine + isoleucine	349 μmol/L (<253)	Organic acidemia	Not identified	Not identified	Not identified	Negative
			Valine	345 μmol/L(<282)					
		0045045	Butyrylcarnitine	0.52 μmol/L (<0.45)					
	3bINP-103	0001992	Urinay organic acid profile	Elevation of 2-hydroxybutiric and 3-OH butyric acid	Organic acidemia	Not identified	Not identified	Not identified	Negative
		0001942	Hyperlactatemia	5.8 (1-3.3 mmol/L)					
		0045045	3-hydroxy-isovalerylcanitine + methylmalonylcarnitine	1.11 μmol/L (<0.83)					

^A^ Patient with a normal chromosomal microarray analysis result. ^B^ No other methodology was applied to the identification of the second pathogenic allele. ^C^ A possible androgenetic/biparental chimerism or genome-wide paternal uniparental disomy is still under study.

**Table 4:** Co-occurrence of two monogenic diseases due to expected, incidental, or secondary findings in the studied patients.

Study Group	Patient ID	HPO	Observed Biochemical Abnormality	1st Disease Detected	Gene Responsible of First Disease	Genotype ^A^	Identified Second Monogenic Disease	Gene Responsible of Second Disease	Genotype ^A^	Type of Finding
1	3bINP-021	12026	Hyperornithinemia	Hyperornithinemia-hyperammonemia-hyperhomocitrullinuria syndrome (OMIM #238970)	***SLC25A15***	NM_014252.4:c.[113_116dup];[113_116dup] or p.[Phe40AspfsTer4];[Phe40AspfsTer4]	Autosomal dominant polydactyly, postaxial, types A1 and B (OMIM #174200)	***GLI3***	**NM_000168.6:c.[3740_3743dup];[3740=] or p.[Cys1249AlafsTer3];[Cys=]**	Expected
		0001987	Hyperammonemia							
	3bINP-054	0001992	Biotinidase deficiency	Biotinidase deficiency (OMIM #253260)	***BTD***	NM_001370658.1:c.468G>T(;)1270G>C or p.(Lys156Asn)(;)(Asp424His)	Autosomal dominant *FGFR2*-related disorder (OMIM *176943)	***FGFR2***	NM_000141.5:c.[923A>G];[923=] or p.[Tyr308Cys];[Tyr=]	Expected
	3bINP-069	0008344	Elevated circulating branched chain amino acid concentration	MSUD type II (OMIM #620699)	***DBT***	NM_001918.5:c.[75_76del];[75_76del] or p.[Cys26TrpfsTer2];[Cys26TrpfsTer2]	Autosomal recessive ATP-binding cassette, subfamily a, member 4 (*ABCA4*)-related disorder (OMIM *601691)	***ABCA4***	NM_000350.3:c.[2453G>A];[2453G>A] or p.[Gly818Glu];[Gly818Glu]	Incidental
	3bINP-074	0410051	3-methylglutaric aciduria	HMG-CoA lyase deficiency (OMIM #246450)	***HMGCL***	NM_000191.3:c.230del(;)31C>T or p.(Val77GlyfsTer16)(;)(Arg11Ter)	Autosomal dominant Lynch syndrome (OMIM #614350)	***MSH6***	**NM_000179.3:c.[2150_2153del];[2150=] or p.[Val717AlafsTer18];[Val=]**	Secondary
	3bINP-082	0000531	Cystinosis	Nephropathic cystinosis (OMIM #219800)	***CTNS***	NM_004937.3:c.[22_23del];[1036_1047del] or p.[Ile8PhefsTer13];[Asp346_Phe349del]	X-linked Alport syndrome type 1 (OMIM #301050)	***COL4A5***	NM_033380.3:c.[3088G>A];[3088=] or p.[Gly1030Ser];[Gly=]	Expected
	3bINP-109	0002156	Homocystinuria	Homocystinuria, B6-responsive and nonresponsive types (OMIM #236200)	***CBS***	NM_000071.3:c.[572C>T];[572C>T] or p.[Thr191Met];[Thr191Met]	Autosomal dominant Fleck corneal dystrophy (OMIM #121850)	***PIKFYVE***	NM_015040.4:c.[853_854del];[853=] or p.[Leu285PhefsTer19];[Leu=]	Incidental
2	3bINP-045	0001943	Hypoglycemia	Autosomal dominant form of Hyperinsulinemic hypoglycemia familial type 1 (OMIM #256450)	***ABCC8***	NM_000352.6:c.[2506C>T];[2506=] or p.[Arg836Ter];[Arg=]	Autosomal dominant *RET*-related disorders, including Multiple endocrine neoplasia (MEN) IIA (OMIM #171400), MEN IIB (OMIM #162300), and familial medullary thyroid carcinoma (OMIM #155240)	***RET***	NM_020975.6:c.[2410G>A];[2410=] or p.[Val804Met];[Val=]	Secondary
	3bINP-047	0045045	Inespecific acylcarnitine alterations	Alpha-methylacetoacetic aciduria (OMIM #203750)	***ACAT1***	NM_000019.4:c.[473A>G];[473A>G] or p.[Asn158Ser];[Asn158Ser]	Autosomal dominant Cardiomyopathy, dilated, type 1G (OMIM #604145)	***TTN***	NM_001267550.2:c.[87470_87471del];[87470=] or p.[Leu29157GlnfsTer6];[Leu=]	Secondary
	3bINP-085	0001943	Hypoglycemia	Autosomal dominant form of Hyperisulinism-hyperammonemia syndrome (OMIM #606762)	***GLUD1***	NM_005271.5:c.[1466C>G];[1466=] orp.[Pro489Arg];[Pro=]	X-linked Glucose-6-phosphate dehydrogenase deficiency (OMIM #300908)	***G6PD***	Hemizygous male for haplotype NM_001360016.2:c.[376A>G;202G>A];[0] or p.[Asn126Asp;Val68Met];[0]	Expected
	3bINP-100	0100651	Hypoglycemia, type I diabetes mellitus	Diarrhea 4, malabsotive, congenital (OMIM #610370)	***NEUROG3***	NM_020999.4:c.[117del];[117del] or p.[Thr40LeufsTer38];[Thr40LeufsTer38]	Autosomal dominant Wagner vitreoretinopathy (OMIM #143200)	***VCAN***	**NM_004385.5:c.[3455C>A];[3455=] or p.[Ser1152Ter];[Ser=]**	Incidental

^A^ Described genotypes considered only pathogenic and likely pathogenic variants. Novel variants are highlighted in bold.

**Table 5:** Decisions taken in medical or nutritional management in the studied patients after WES by categories: (1) modification of the initial treatment, (2) continuation of the initial treatment, or (3) no treatment was provided before or after WES.

**Decision**	**Cause of Change or Mantainance**	**Patient ID**	**Initial Biochemical Diagnosis**	**Final WES Diagnosis**	**Initial Medical or Nutritional Management**	**Final Medical or Nutritional Management**
**(1) Modification of the initial treatment (n = 18)**	Discordance between initial and final diagnosis	3bINP-001	Unspecific propionylcarnitine elevation; dysmorphological syndrome	Negative + Coffin-Siris syndrome type 10 (OMIM #618506) ^A^✦	B12 vitamin supplementation	Gradually B12 vitamin suspension as blood B12 levels normalized, plus closer monitoring by the orthopedics, cardiology, otorhinolaryngology, and neurology services.
		3bINP-041	Hyper beta-alaninemia	Negative + Autosomal dominant lissencephaly type 1 (OMIM #607432) ^B^✦	B6 vitamin supplementation	Gradually B6 vitamin suspension, plus closer monitoring by neurology service.
	A second disease found	3bINP-045	Hypoglycemia	AD Hyperinsulinemic hypoglycemia familial 1 + Autosomal dominant *RET*-related disorder (secondary finding)	Fasting avoidance	Continue with initial medical management, plus closer monitoring by oncology service, segregation analysis, and genetic counseling as the mother resulted heterozygous for *RET* pathogenic genotype
		3bINP-047	Unspecific acylcarnitine alterations	Alpha-methylacetoacetic aciduria + Autosomal dominant Cardiomyopathy, dilated, type 1G (secondary finding)	None	Initiation of nutritional treatment, plus referal to cardiology service for closer monitoring.
		3bINP-069	MSUD	Maple syrup urine disease + *ABCA4*-related retinal distrophy (incidental finding)	Branched chain amino acids restricted diet	Continue with initial nutritional management, plus close monitoring by the ophthalmology service.
		3bINP-074	3-hydroxy-3-methylglutaric aciduria	3-hydroxy-3-methylglutaric aciduria + Autosomal dominant *MSH6*-related Lynch syndrome (secondary finding)	Nutritional treatment, leucine and lipid restricted diet, carninite supplementation	Continue with initial nutritional management, plus closer monitoring by oncology service, segregation analysis, and genetic counseling as the father resulted heterozygous for *MSH6* pathogenic genotype.
		3bINP-085	Hypoglycemia	Hyperinsulinism hyperammonemia syndrome + X-linked glucose-6-phosphate dehydrogenase deficiency (expected finding)	Fasting avoidance. Diet high in complex carbohydrates such as corn starch, along with the recommended daily protein intake	Continue with initial nutritional management, plus diazoxide prescription, genetic counseling on risks of hemolytic anemia, and closer medical follow-up.
		3bINP-100	Hypoglycemia, diabetes mellitus type 1	Congenital diarrhea type 4 malabsorptive + Autosomal dominant Wagner vitreoretinopathy (incidental finding)	Fasting avoidance, insulin	Continue with initial medical management, plus close monitoring by the ophthalmology and gastroenterology services.
		3bINP-109	Homocystinuria	Homocystinuria + Autosomal dominant Fleck corneal dystrophy (incidental finding)	Methionine restricted diet, betaine, B6 vitamin and folic acid supplementation, and monthly intake of B12 vitamin	Continue with initial nutritional management, plus close monitoring by the ophthalmology service.
	Initial unspecific diagnosis + negative WES	3bINP-006	Suspicion of a FAOD for subtle elevation of tetradecanoylcarnitine and urinary excretion of adipic, suberic and sebacic acids	Negative	Long chain fatty acid restricted diet and medium-chain triglycerides supplementation	Gradual release from the nutritional management and redirection of the diagnostic approach
		3bINP-016	Suspicion of hypermethioninemia due to subtle elevation of blood methionine and homocysteine	Negative	Methionine restricted diet	Gradual release from the nutritional management and redirection of the diagnostic approach
		3bINP-024	Suspicion of UCD because of hyperammonemia	Negative	Protein restricted diet, sodium benzoate and L-carnitine supplementaion	Gradual release from the nutritional management and redirection of the diagnostic approach
		3bINP-026	Suspicion of hyperalaninemia because of elevation of blood alanine	Negative	Ketogenic diet	Gradual release from the nutritional management and redirection of the diagnostic approach
		3bINP-031	Suspicion of hypermethioninemia because of 4-fold elevation of methionine and 3-fold elevation of Met/Phe ratio	Negative	Methionine restricted diet	Gradual release from the nutritional management and redirection of the diagnostic approach
		3bINP-050	Suspicion of organic acidemia because of subtle propionylcarnitine elevation	Negative	B12 vitamin supplementation	Gradual release from the nutritional management and redirection of the diagnostic approach
		3bINP-056	Suspicion of FAOD due to unspecific elevation of blood long chain acylcarnitines	Negative	Fasting avoidance and long chain fatty acid restricted diet	Gradual release from the nutritional management and redirection of the diagnostic approach
		3bINP-063	Succinyl-CoA:3-oxoacid-CoA transferase deficiency due to elevated excretion of 3-hydroxybutiric and acetoacetic acids	Negative	Isoleucine restricted diet	Gradual release from the nutritional management and redirection of the diagnostic approach
		3bINP-072	Suspicion of GSD due to positive liver biopsy	Negative	Fasting avoidance. Diet high in complex carbohydrates such as corn starch, along with the recommended daily protein intake	Gradual release from the nutritional management and redirection of the diagnostic approach
**(2) Continuation of initial treatment (n = 5)**	Monoallelic genotype found	3bINP-036	Organic acidemia for presence of 3-hydroxy-isovaleryl carnitine + methylmalonyl carnitine	Only one variant in *HLCS* gene	Biotin supplementation	Maintainance of biotin supplementation
		3bINP-052	MCAD deficiency for the elevation of hexanoyl, octanoyl, and decanoyl carnitines	Only one variant in *ACADM*	Fasting avoidance	Maintainance of fasting avoidance
	Genotype constituted of two VUS variants	3bINP-076	MSUD for remarkable blood elevation of branched chain amino acids and elevated excretion of branched chain keto acids in urine	Presence of two VUS variants in *DBT*	Branched chain amino acids restricted diet	Maintainance of branched chain amino acids restricted diet
		3bINP-089	Galactosemia for blood elevation of galactose, and normal activity of galactose-1P-uridyl transferase	Presence of two VUS variants in *GALK1*	Galactose restricted diet	Maintainance of galactose restricted diet
		3bINP-094	MSUD for remarkable blood elevation of branched chain amino acids and alloisoleucine, and elevated excretion of branched chain keto acids in urine	Presence of two VUS variants in *DBT*	Branched chain amino acids restricted diet	Maintainance of branched chain amino acids restricted diet
**(3) No specific treatment was provided before or after WES (n = 9)**	Not confirmated unspecific biochemical findings	3bINP-007	Suspicion of hyperprolinemia due to subtle elevation of blood proline	Negative	None	Redirection of the diagnostic approach
		3bINP-009	Suspicion of a carbohydrate disorder due to hypoglycemia and hyperinsulinism	Negative	None	Redirection of the diagnostic approach
		3bINP-034	Suspicion of FAOD due to subtly altered acylcarnitines profile	Negative	None	Redirection of the diagnostic approach
		3bINP-038	Suspicion of MSUD because of subtle elevation of branched chain amino acids	Negative	None	Redirection of the diagnostic approach
		3bINP-039	Suspicion of hyperprolinemia due to subtle elevation of blood proline	Negative	None	Redirection of the diagnostic approach
		3bINP-051	Suspicion of FAOD due to unspecific altered acylcarnitines profile	Negative	None	Redirection of the diagnostic approach
		3bINP-070	Suspicion of MSUD because of subtle elevation of branched chain amino acids	Negative	None	Redirection of the diagnostic approach
		3bINP-093	Suspicion of MSUD vs organic acidemia for subtle elevation of branched chain amino acids and butyrylcarnitine	Negative	None	Redirection of the diagnostic approach
		3bINP-103	Suspicion of organic acidemia for subtle elevation of 3-hydroxy-isovaleryl carnitine + hyperlactatemia	Negative	None	Redirection of the diagnostic approach

^A^ *SOX4* genotype: NM_003107.3(SOX4):c.[1061C>A];[=] or p.[Ser354*];[=]. ^B^ PAFAH1B1 genotype: NG_009799.1(NM_000430.4):c.[116_117+2dup];[=] or p.[?];[=]. ✦ Syndromic entities not related to IEiM. Abbreviations: MSUD, maple syrup urine disease; FAOD, fatty acid oxidation disorder; GSD, glycogen storage disease; UCD, urea cycle disorder; MCAD, medium-chain acyl-CoA dehydrogenase deficiency.

## Data Availability

Publicly available datasets were analyzed in this study. The data can be found here: ClinVar: https://www.ncbi.nlm.nih.gov/clinvar/, accessed on 20 May 2024; dbSNP: https://www.ncbi.nlm.nih.gov/snp/, accessed on 20 May 2024; Genome Aggregation Database (gnomAD) v.2.1.1: https://gnomad.broadinstitute.org/, accessed on 20 May 2024; Leiden Open Variation Database (LOVD) v.3.0: https://www.lovd.nl/, accessed on 21 June 2024; Online Mendelian Inheritance in Man (OMIM): https://www.omim.org/, accessed on 20 May 2024; The National Center for Biotechnology Information (NCBI): https://www.ncbi.nlm.nih.gov/gene, accessed on 20 May 2024, and The Human Gene Mutation Database (HGMD): https://www.hgmd.cf.ac.uk/, accessed on 20 May 2024). The data presented in this study are available upon reasonable request from the corresponding author. The clinical and molecular data of patients and their relatives are not publicly available due to restrictions to preserve their confidentiality, which was part of the signed informed consent of each patient. All the herein reported clinically relevant genetic variants along with the available deidentified phenotypic data were submitted to the publicly available database LOVD v.3.0, Leiden Open Variation Database (https://www.lovd.nl/, accessed on 21 June 2024).

## Supplementary Materials

The following supporting information can be downloaded at: https://www.mdpi.com/article/10.3390/ijms252111722/s1.
